# RIP-seq of BmAgo2-associated small RNAs reveal various types of small non-coding RNAs in the silkworm, *Bombyx mori*

**DOI:** 10.1186/1471-2164-14-661

**Published:** 2013-09-28

**Authors:** Zuoming Nie, Fang Zhou, Dan Li, Zhengbing Lv, Jian Chen, Yue Liu, Jianhong Shu, Qing Sheng, Wei Yu, Wenping Zhang, Caiying Jiang, Yuhua Yao, Juming Yao, Yongfeng Jin, Yaozhou Zhang

**Affiliations:** 1 College of Life Sciences, Zhejiang Sci-Tech University, Hanghzou 310018, China; 2 Zhejiang Economic & Trade Polytechnic, Hangzhou 310018, China; 3 College of Materials and Textile, Zhejiang Sci-Tech University, Hangzhou 310018, China; 4 College of Life Sciences, Zhejiang University, Hangzhou 310058, China

**Keywords:** *Bombyx mori*, BmAgo2-associated small RNAs, RIP-seq, tRFs, BmNPV

## Abstract

**Background:**

Small non-coding RNAs (ncRNAs) are important regulators of gene expression in eukaryotes. Previously, only microRNAs (miRNAs) and piRNAs have been identified in the silkworm, *Bombyx mori*. Furthermore, only ncRNAs (50-500nt) of intermediate size have been systematically identified in the silkworm.

**Results:**

Here, we performed a systematic identification and analysis of small RNAs (18-50nt) associated with the *Bombyx mori* argonaute2 (BmAgo2) protein. Using RIP-seq, we identified various types of small ncRNAs associated with BmAGO2. These ncRNAs showed a multimodal length distribution, with three peaks at ~20nt, ~27nt and ~33nt, which included tRNA-, transposable element (TE)-, rRNA-, snoRNA- and snRNA-derived small RNAs as well as miRNAs and piRNAs. The tRNA-derived fragments (tRFs) were found at an extremely high abundance and accounted for 69.90% of the BmAgo2-associated small RNAs. Northern blotting confirmed that many tRFs were expressed or up-regulated only in the BmNPV-infected cells, implying that the tRFs play a prominent role by binding to BmAgo2 during BmNPV infection. Additional evidence suggested that there are potential cleavage sites on the D, anti-codon and TψC loops of the tRNAs. TE-derived small RNAs and piRNAs also accounted for a significant proportion of the BmAgo2-associated small RNAs, suggesting that BmAgo2 could be involved in the maintenance of genome stability by suppressing the activities of transposons guided by these small RNAs. Finally, Northern blotting was also used to confirm the *Bombyx* 5.8 s rRNA-derived small RNAs, demonstrating that various novel small RNAs exist in the silkworm.

**Conclusions:**

Using an RIP-seq method in combination with Northern blotting, we identified various types of small RNAs associated with the BmAgo2 protein, including tRNA-, TE-, rRNA-, snoRNA- and snRNA-derived small RNAs as well as miRNAs and piRNAs. Our findings provide new clues for future functional studies of the role of small RNAs in insect development and evolution.

## Background

Small non-coding RNAs (ncRNAs) are important regulatory molecules that regulate the expression of target genes by affecting mRNA stability, protein translation and chromosome modification. There are three main classes of small RNAs: microRNAs (miRNAs), small-interfering RNAs (siRNAs) and Piwi-interacting RNAs (piRNAs). Mature miRNAs are a class of ~22nt small RNA molecules that bind to complementary sequences in target genes and regulate gene expression by modulating mRNA translation or stability [1]. miRNAs have been implicated in many cellular processes, including immune mechanisms of biological molecules, stem cell differentiation, cell signal transduction, tumorigenesis, neuronal development and apoptosis [1,2]. siRNAs are a class of double-stranded RNA molecules between 20 and 25nt in length. They are involved in many pathways and regulate the expression of target genes via complementary binding sites [3]. siRNAs are also known to play an important role in antiviral mechanisms and in shaping the chromatin structure of the genome [4-6]. Recently, many endogenous siRNAs were identified that function in transcriptional silencing [7-9]. piRNAs are a class of small RNAs associated with PIWI family proteins. The biogenesis of piRNAs is related to a process called the Ping-pong cycle [10,11]. These small RNAs have been found mainly in stem cells and germ cells, and they are thought to play an essential role in germline development, stem cell renewal, transposon silencing and epigenetic regulation [12-15]. Due to the recent development and application of high-throughput sequencing, many novel small RNAs have been identified, including tRNA-, rRNA-, snoRNA- and snRNA-derived small RNAs [16-21]. tRNAs are not very stable and, under stress, can be sequence-specifically cleaved into different sized fragments termed "tRNA-derived fragments," "tRFs" or "sitRNAs." These fragments could be a novel class of small RNAs. Because they associate with the Ago2 protein, it seems likely that they play a role similar to siRNAs in RNAi silencing [16,17].

The silkworm, *Bombyx mori*, is an important economic insect and has become a model organism for the study of the biochemistry, molecular genetics and genomics of *Lepidoptera* insects [22,23]. Previous studies on small ncRNAs in the silkworm have focused on miRNAs and piRNAs. Our group was the first to provide a large-scale identification of miRNA genes in *Bombyx mori*[24]. Recently, a large number of new miRNAs have been identified using deep sequencing [25-29] and their potential functions were predicted through an analysis of expression profiling performed at various developmental stages and in various tissues of the silkworm [26,30-33]. The miRBase database (http:http://www.mirbase.org/, Release 19.0) has published 567 *Bombyx* miRNAs. piRNAs have also been well characterized in the silkworm. Kawaoka *et al.* analyzed the biogenesis of piRNAs, which could exert an important genomic defense against transposons in the silkworm genome [34-40]. However, less work on siRNAs in the silkworm has been performed, and only 788 potential transposable element (TE)-associated siRNAs have been identified by deep sequencing techniques [18]. In addition, intermediated-sized ncRNAs (50-500nt) have been systematic identified in the silkworm, including 141 snoRNAs, six snRNAs and 38 unclassified ncRNAs [41]. Based on the recent identification of an increasing number of small RNAs, it seems likely that many novel small RNAs remain to be discovered in *Bombyx mori*.

To execute their functions, small RNAs must be incorporated into the RNA-induced silencing complex (RISC). Once incorporated, the small RNAs guide RISC to its complementary targets and thereby regulate the expression of the targets at the transcriptional or translational levels [42,43]. The Argonaute (Ago) protein is a central protein component of RISC and exerts specialized functions on the miRNA and RNAi pathway [44-46]. The mammalian Argonaute family has four members, but only Ago2 possesses slicer endonuclease activity [46,47]. The isolation of RNAs associated with Ago proteins has become an important means for the discovery and functional assessment of novel small RNAs [18,21,48-52] and for the target genes of miRNAs [53,54]. *Bombyx mori* Argonaute2 (BmAgo2, GenBank accession: NM_001043530.2) belongs to the Ago family and is an ortholog of *Drosophila* Argonaute2, which contains the conserved amino acid residues D965, D1037 and H1173. These conserved residues are critical for the nuclease activity of Ago2. Previous reports have shown that in silkworm infected with *Bombyx mori* nucleopolyhedrovirus (BmNPV), BmAgo2 expression is up-regulated, which could be related to the RNA silencing machinery involved in DNA virus infection in insects [55,56]; however, this mechanism will require further study.

Ago proteins are key components of the siRNA and miRNA pathway and are indispensable binding proteins for the function of many other small RNAs. Therefore, the isolation of Ago-associated small RNAs is an important approach for identifying functional small RNAs [18,19,21]. In this study, we extracted the total small RNAs (18-50nt) that associated with BmAgo2 protein using the RNA immunoprecipitation (RIP) method. Subsequent deep sequencing, bioinformatics analysis and Northern blotting were used to identify various types of small RNAs associated with the BmAgo2 protein, including tRNA-, TE-, rRNA-, snoRNA- and snRNA-derived small RNAs as well as miRNAs and piRNAs. Further analysis revealed that these small RNAs possess novel characteristics.

## Results

### RIP of BmAgo2 from BmN cells infected with recombinant BmNPV virus

**Figure 1:**
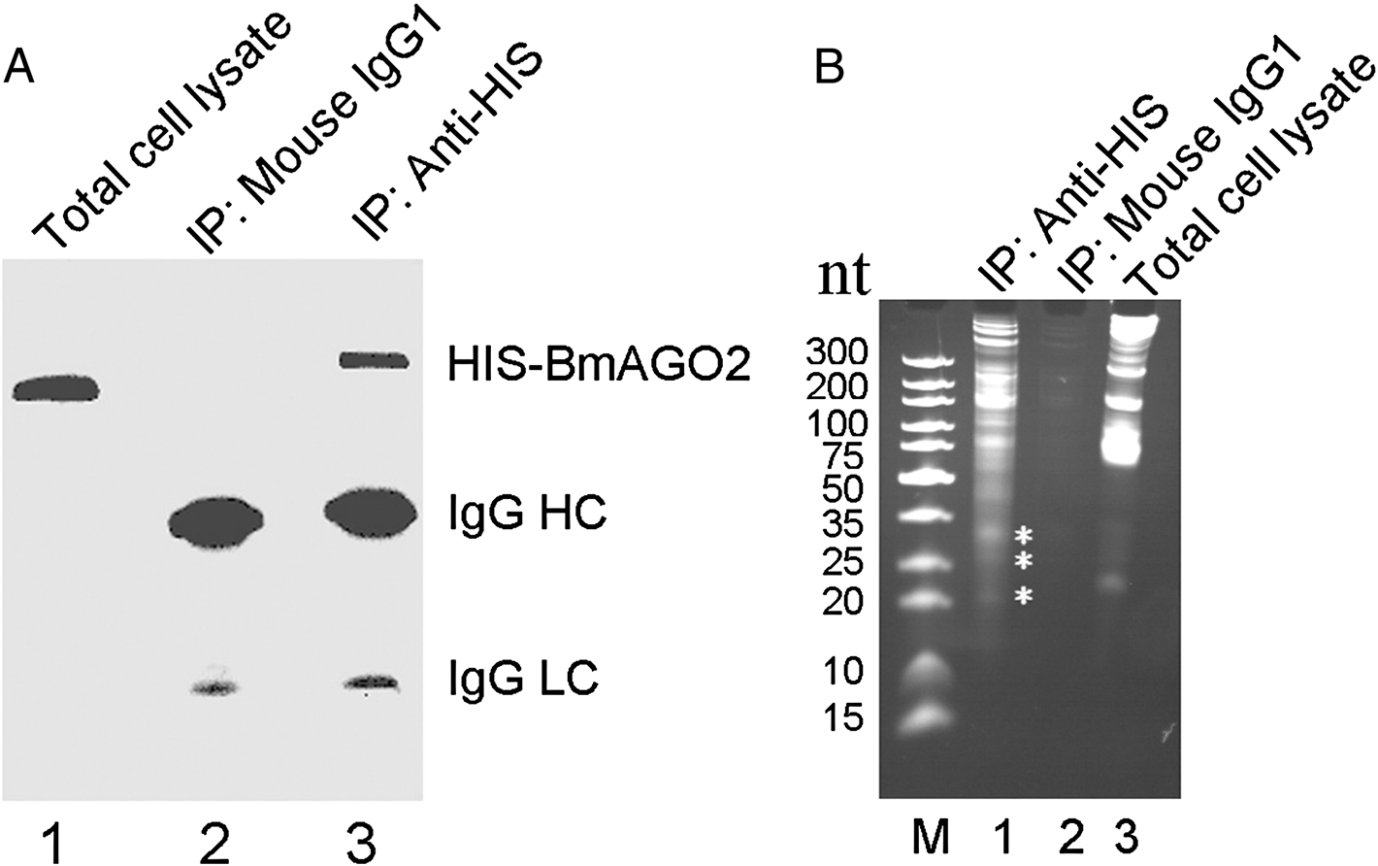
**RIP for the isolation of BmAgo2-associated RNAs from BmN cells. (A)** Co-Immunoprecipitation of HIS-BmAgo2 using HIS monoclonal antibody. IP was validated by Western blotting using a HIS monoclonal antibody. BmAgo2 was successfully expressed in BmN cells infected with recombinant BmNPV virus (lane 1) and was pulled down when the HIS antibody was used (lane 3). Mouse IgG1 was used as a negative control and indeed revealed no IP of BmAgo2 (lane 2). **(B)** Co-immunoprecipitated RNAs analyzed by PAGE. Compared to the total RNAs of the BmN cells (lane 3), the HIS monoclonal antibody pull-down RNAs showed an obvious RNA smear ranging from 20 bp to 50 bp (lane 1). The negative control mouse IgG1 did not immunoprecipitate visible RNA amounts (lane 2). Lane M shows a DNA size marker. Asterisks indicate the visible bands for the ~20nt, ~27nt and ~33nt small RNA species, respectively.

Small RNAs and their targets bind the Ago-containing RISC complexes, in which the Ago proteins form stable Ago ribonucleoproteins that can be biochemically analyzed [53,57,58]. The Ago-protein-binding small RNAs can be isolated by RIP [59,60]. In a previous work, *BmAgo2* was fused with a HIS tag and was successfully expressed using the Baculovirus Bacmid system harboring the ie1 promoter enhanced with a hr5 enhancer [61]. The recombinant viruses were then harvested at 20 hrs post infection, and HIS-BmAgo2 could be detected at a high level by Western blotting with a HIS monoclonal antibody (Additional file 1: Figure S1). The HIS monoclonal antibody (mouse anti-(his)6, Roche) was used to immunoprecipitate HIS-BmAgo2-containing RISC from the total cell lysate of the infected BmN cells. The approximately 120 kDa HIS-BmAgo2 was identified by Western blotting in the total cell lysate and HIS-BmAgo2 IP fraction but was absent in the IP fraction of the negative control (Figure 1A). The co-immunoprecipitated BmAgo2-bound RNAs were extracted and analyzed by PAGE. Interestingly, the RNA collected via the HIS-BmAgo2-specific monoclonal antibody pull-down showed a much more dense RNA smear than the total RNAs of the BmN cells, ranging from 18 bp to 50 bp (Figure 1B). In this region, three visible bands of ~20nt, ~27nt and ~33nt accumulated, indicating that small RNAs may be recruited by the BmAgo2 complexes and may function together with the BmAgo2 protein. The negative control IgG1 from the mouse did not immunoprecipitate visible amounts of RNAs (Figure 1B), indicating that the BmAgo2-associated RNA signal was specific. Taken together, our results demonstrate that BmAgo2 can bind many small RNAs that can be biochemically isolated.

### Sequencing and analysis of a BmAgo2-associated small RNA library

Ago proteins have an important role in small RNA pathways and mediate the interaction between small RNAs and their targets. The resolution of Ago-associated small RNAs showed a significant landscape of Ago proteins and their binding to small RNAs [18,19,21,48-50]. To characterize the small RNAs that associate with the BmAgo2 protein in *Bombyx mori*, the small RNA population associated with this protein in BmN cells was extracted from the Ago immunoprecipitated complex. Small RNAs between 18nt and 50nt were separated by PAGE and were then subjected to library construction and deep sequencing.

The high throughput sequencing yielded a total of 813,702 unique reads, representing 11,691,441 high-quality total reads of between 1 and 5,731,905 copies. The reads were then mapped to the *Bombyx mori* genome. Of the 813,702 unique reads, only 538,567 mapped to the reference genome when mismatches were not allowed, accounting for 66.19% of the total unique reads and showing no preference for the distribution between the chromosomes; however, when a maximum of two mismatches were allowed, 722,610 unique reads mapped to the reference genome, accounting for 88.81% of the total unique reads, suggesting that many mutations, base edits or SNPs exist in these small RNAs (Additional file 1: Figure S2A). Thus, 11.19% of the total unique reads did not match the genome, which may also be due to possible gaps in the sequenced *Bombyx mori* genome [62,63]. In addition, the reference genome was derived from male larvae and therefore did not contain sequences from the W chromosome [62,63]. The silkworm W chromosome was a source of small RNAs in *Bombyx*, such as the female-enriched piRNAs [36]. Of the 91,092 unmapped unique reads, 9,206 mapped to contig sequences from the W chromosome, which form a small portion of the whole W chromosome. Additionally, 218 unique reads derived from the BmNPV genome were identified.

**Figure 2:**
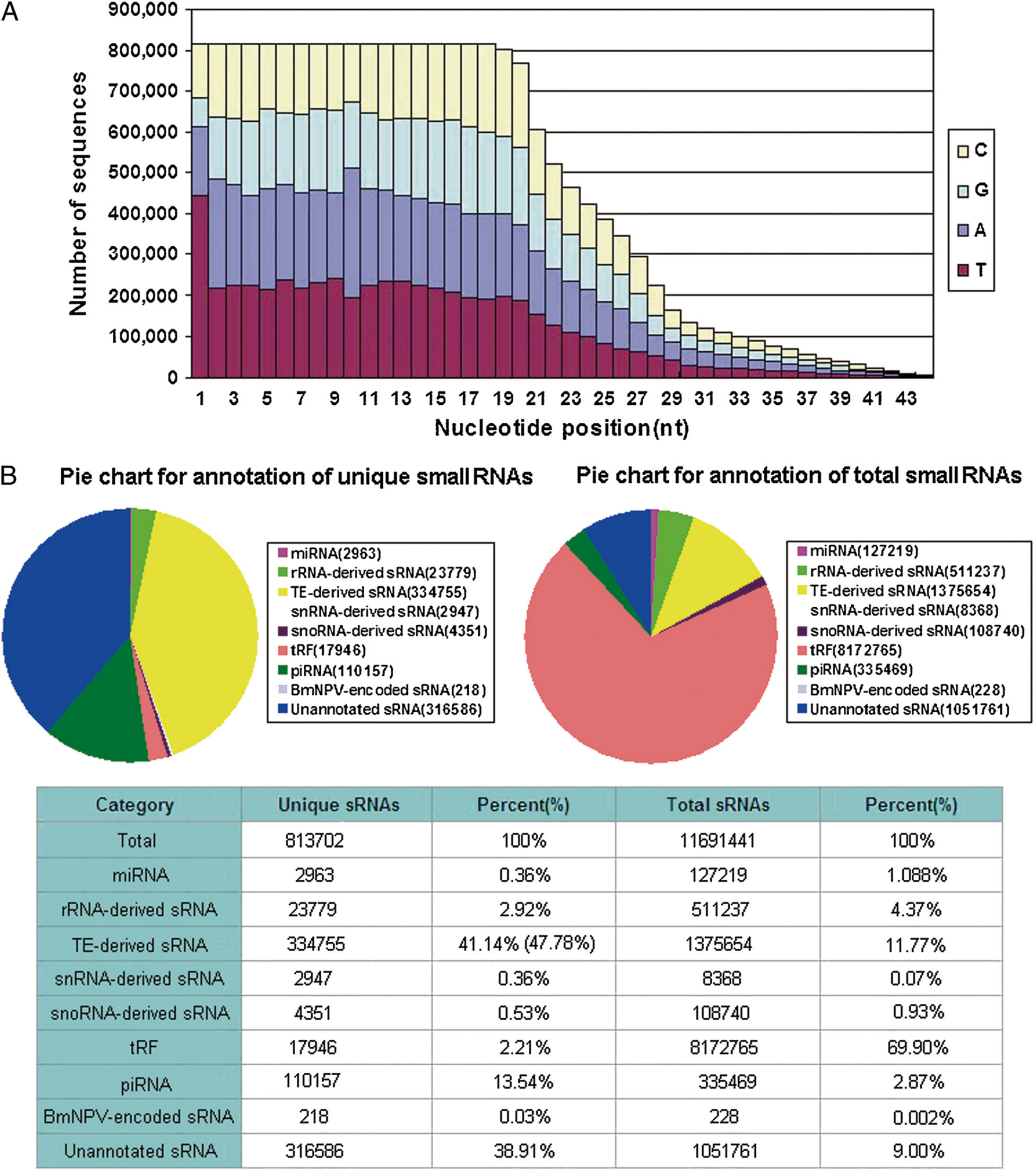
**Characterization of BmAgo2-associated small RNAs. (A)** Nucleotide compositions of unique small RNAs. BmAgo2-associated small RNAs displayed a strong bias towards "U" at the 5′ end (54.47%). **(B)** Classification and annotation of small RNAs using the bioinformatics pipeline described in the Materials and Methods. tRFs were recruited by the BmAgo2 protein at an extremely high abundance, and the unannotated small RNAs comprised many diverse sequences with a low expression abundance. Of the 110,157 piRNA candidates, 54033 were derived from TEs, accounting for 6.64% of the total unique small RNAs. Thus, the actual percent of TE-derived sRNAs was 47.78% (41.14% + 6.64%). sRNAs: small RNAs.

We investigated the length distribution of the obtained BmAgo2-associated small RNAs. The reads showed a multimodal length distribution, with three peaks at ~20nt, ~27nt, and ~33nt (Additional file 1: Figure S2B). These sizes were consistent with the sizes of the bands separated by PAGE (Figure 1B). The length distribution of the BmAgo2-associated small RNAs was different than that of the SjAgo2-associated small RNAs, which showed only one peak at 21nt [18]. The number of 32nt and 33nt small RNAs in the BmAgo2-associated total small RNA population decreased significantly when the unique sequences were analyzed, but the number of ~20nt and ~27nt small RNAs did not change much. This finding suggests that the BmAgo2-associated ~20nt and ~27nt small RNAs comprised a large number of diversified sequences with low expression abundance, whereas the expression of the ~33nt small RNAs occurred at an extremely high level (Additional file 1: Figure S2B and C, Additional file 2: Table S1). Additionally, we observed a strong preference for the first 5′ nucleotide to be "U" in the unique BmAgo2-associated small RNA sequences (accounting for 54.7% of the unique small RNA sequences) and "A" was similarly enriched at position 10 (Figure 2A), consistent with previous reports [19,34,35,64,65].

The obtained small RNAs were then classified and annotated using the bioinformatics pipeline described in the Materials and Methods. The results showed that "housekeeping" non-coding RNAs, such as tRNAs, rRNAs, snRNAs and snoRNAs, could produce BmAgo2-associated small RNAs. Thus, various types of small ncRNAs were identified among the BmAgo2-associated small RNAs, including TE-, tRNA-, rRNA-, snoRNA and snRNA-derived small RNAs as well as miRNAs and piRNAs (Figure 2B). In addition, the 218 BmNPV-derived unique reads might be novel BmNPV-encoded small RNA species. The unique small RNA species were dominated by TE-derived small RNAs, which accounted for 47.78% of the unique small RNAs, but the total small RNAs were overwhelmingly dominated by tRFs, which accounted for 2.21% of the unique small RNAs and 69.90% of the total small RNAs. We also identified many small RNAs without clear structural features or functions, which we defined as unannotated small RNAs. The unannotated small RNAs comprised a large number of diverse sequences with low expression and accounted for 38.91% of the unique small RNAs and 9.00% of the total small RNAs.

### tRFs

With the development of molecular cloning for the identification of cellular RNA fragments and deep sequencing, a number of classes of small RNAs have been identified recently, including a novel class of small RNAs in eukaryotes. This group of molecules, termed tRFs, comprises RNA fragments derived from either tRNAs or tRNA precursors [16,17,20,66]. tRFs were the most abundant small RNA identified from the deep sequencing of the BmAgo2-associated small RNAs, accounting for 69.90% of the total small RNAs. However, tRFs accounted for only 2.21% of the unique small RNAs, implying that an extremely high abundance but a low species number of tRFs are recruited by the BmAgo2 protein (Figure 2B).

**Figure 3:**
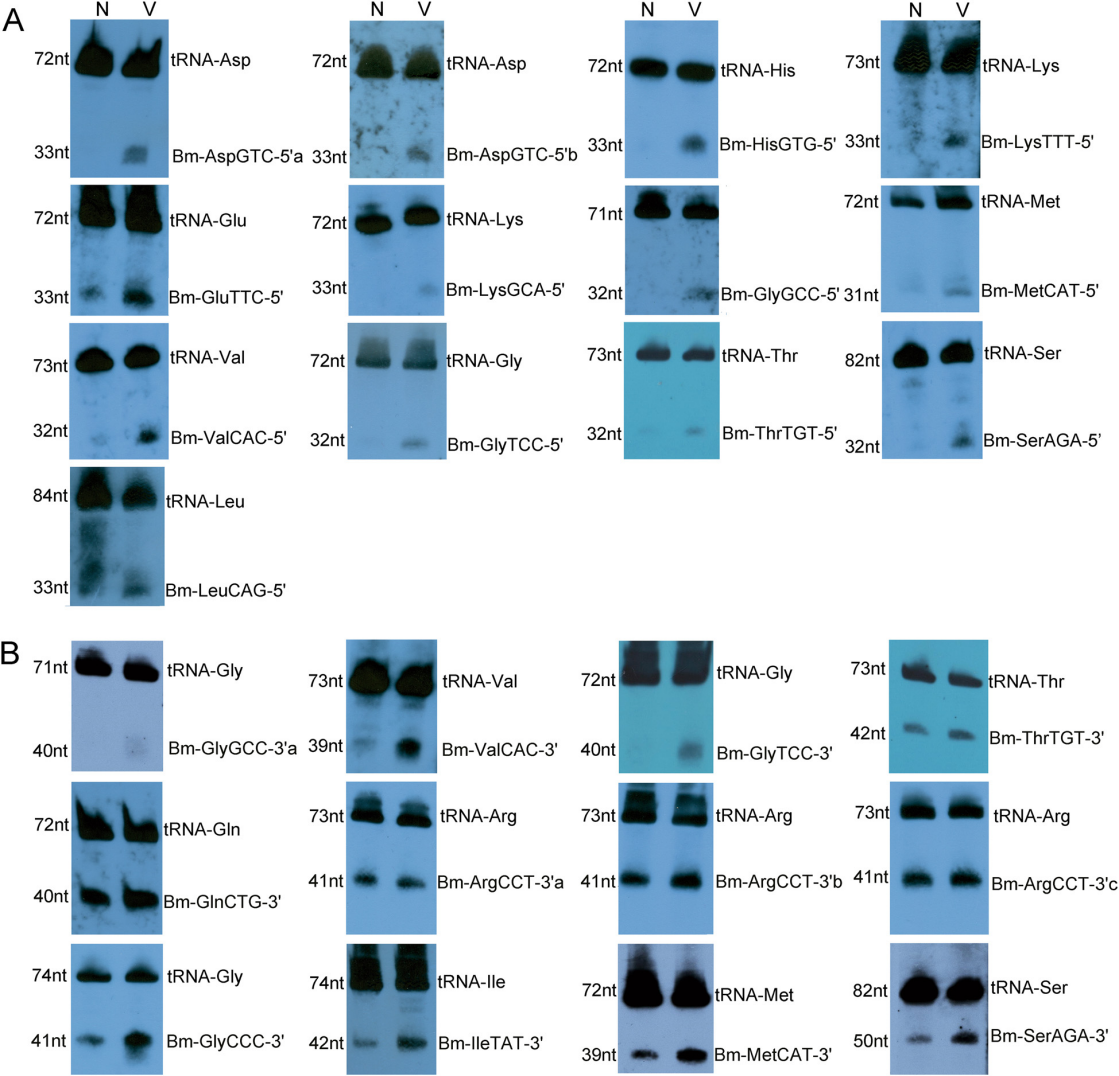
**Identification of tRFs by Northern blotting. (A)** 5′tRFs were identified by Northern blotting with a length of ~33 bp. The majority of the identified 5′tRFs were generated only in BmNPV-infected cells, implying a BmNPV-related role for these small RNAs. **(B)** 3′tRFs were identified by Northern blotting with a length of ~40 bp. However, the majority of the identified 3′tRFs were generated in both the normal cells and in the BmNPV-infected cells. The host tRNAs were also identified along with the tRFs. N: normal BmN cells; V: BmNPV-infected BmN cells.

To further confirm the expression of the tRFs and to determine their size, we used Northern blotting to analyze the selected tRFs. The tRFs with read copy numbers of no less than 150 and redundancy removal were selected. In addition, the other selected tRFs were derived from the same host tRNA that generate the previously selected tRFs. Our results showed that *Bombyx* tRNAs could generate small RNAs, which were derived mainly from the 5′ or 3′ termini of the tRNA (termed the 5′tRF or 3′tRF, respectively). There were two types of 5′tRF molecules. One was a ~33nt 5′tRF, which was located at the 5′ termini of the host, tRNA and produced via cleavage of the anti-codon loop by a certain nuclease (Figure 3A and Additional file 2: Table S2). The other was a truncated 18nt 5′tRF molecule, which was produced by further cleavage of the ~33nt 5′tRF at the D loop; this 5′tRF molecule could not be detected using Northern blotting but could be sequenced at a high read copy number (Bm-SerAGA-5′T and Bm-LeuCAG-5′T, Additional file 2: Table S2). The 3′tRFs also comprised two types of molecules. One was an ~40nt small RNA that was located at the 3′ termini of the host tRNA and was produced through the cleavage of the anti-codon loop (Figure 3B and Additional file 2: Table S2). The second was a 21nt 3′trailer tRF small RNA located between the anti-codon and TψC loop of the host tRNA (tRNA 3′trailer) that was produced by further cleaving the ~40nt 3′tRF at the TψC loop. The 3′trailer tRF could not be detected using Northern blotting but could be sequenced with a very high read number (Bm-MetCAT-3′T, Bm-GlnCTG-3′T, Bm-GlyCCC-3′T and Bm-IleTAT-3′T, Additional file 2: Table S2). A GTTC motif appeared in the 3′termini of the 21nt 3′trailer tRFs due to a conserved cleavage site at the end of the GTTC motif in the TψC loop (Additional file 1: Figure S3). The asymmetric source of tRFs from the tRNAs showed that the tRFs are not random by-products of tRNA biogenesis and degradation but are instead an abundant and novel class of small RNAs.

Interestingly, *Bombyx* tRFs seem to play a prominent role in the response of *Bombyx mori* to BmNPV. Many tRFs showed very high expression levels in the BmNPV-infected BmN cells (Figure 3). Among these tRFs, some exhibited obviously up-regulated expression in the BmNPV-infected BmN cells, while others were expressed only in the BmNPV-infected BmN cells (Figure 3A). These results suggest that the tRFs are important stress factors and might have an important function during baculovirus infection. Importantly, almost all of the detected 5′tRFs were expressed in only the infected BmN cells; thus these tRFs are most likely produced under the stress of viral infection. The levels of the corresponding tRNAs were not increased in the infected cells, indicating that it is the processing to tRFs that is changed in the infected cells. Surprisingly, the ~33nt 5′tRF molecules accounted for an overwhelming proportion of the total small RNAs bound to BmAgo2 (Figure 2B and Additional file 2: Table S2) in the infected cells, especially Bm-AspGTC-5′a, which accounted for 49.03% (5,731,905/11,691,441) of the total BmAgo2-associated small RNAs. The biogenesis of tRFs under conditions of virus stress provides an important piece of evidence for their functional role in the cell. A previous report showed that tRFs are endogenously associated with Ago2 and are able to guide Ago2 to cleave its target RNA [16]. BmAgo2 could thus recruit large numbers of tRFs, forming a RNP complex similar to RISC and thereby regulating the expression of tRF-guided target genes. The precise functions of tRFs remain unknown and should be further studied.

### Transposable-element derived small RNAs and piRNAs

To identify small RNAs derived from TEs (TE-derived small RNAs) associated with BmAgo2, the unannotated reads were mapped to the *Bombyx mori* TEs. Using this method, we obtained 388,788 unique small RNA reads derived from the TEs, which accounted for 47.78% of the total unique reads (388,788/813,702); 56.48% of these mapped to multiple locations (219,578/388,788). This finding suggests that the population of TE-derived small RNAs is significantly higher than that of the small RNAs derived from miRNAs, tRNAs, rRNAs, snoRNAs and snRNAs in the BmAgo2-associated small RNA population. The length distribution ranged from 18nt to 44nt and peaked at 20nt, with a secondary peak at 21nt, implying that the main siRNA proportion comprised TE-derived small RNAs (Additional file 2: Table S3). TEs are currently regarded as one of the principal forces driving genome diversity and evolution but must be under appropriate control to maintain genome integrity [67,68]. In *Bombyx mori*, the repetitive sequences are estimated to account for 43.6% of the total sequence [62,63], implying that active functions and regulation of TEs must be in action. We further investigated the TE-derived small RNAs restricted to particular classes of transposons. Of the obtained 2320 TEs, 2080 could generate small RNAs at an abundance between 1 and 24,484. The majority of these small RNAs were derived mainly from LTR and LINE retrotransposons, similar to the small RNAs associated with sjAgo2 [18]. The top 25 TE-derived small RNA clusters and their mapping reads are summarized in Table 1 and Additional file 2: Table S4. The top 25 TEs consisted of 11 LTR and 9 LINE TEs; the only ClassII TE was BmpiggyBac-MER85, a widely studied transposon in insects. A previous report showed that LINE bm1645 strongly and preferentially generated antisense small RNAs [18]. We further observed that BmAgo2 could associate with small RNAs derived from bm1645 including 12539 antisense small RNAs and 1732 sense small RNAs. The other TEs preferentially produced antisense small RNAs, including SART1, bm447 and bm1770 (antisense/sense > 2). Only Gypsy-20_BM-I preferentially produced sense small RNAs (sense/antisense >2).

**Table 1:** Top 25 TE-derived small RNAs associated with BmAgo2

**Name of TEs**	**TE types**	**Length**	**piRNAs**		**Other small RNAs**	
			**Sense**	**Antisense**	**Sense**	**Antisense**
Gypsy-9_BM(Aquila)-I	LTR/ Gypsy	7716	621(2803)	692(5297)	12022(57722)**	11149(52894)
bm1866	LTR/Pao	6465	576(1668)	613(1988)	6921(27652)	7048(28122)
bm1645	LINE/R1	13595	42(54)	633 (4794)	1690(3400)	11906(220080)
bm1456	LTR/Pao	8702	570(2359)	671(2944)	4135(22334)	3842(20498)
L09635	LTR/Pao	4791	376(1123)	408(1644)	4043(23398)	4177(18148)
AB480244.1(BMC1) *	LINE/Jockey	25948	474(1757)	359(821)	3598(26788)	2713(8313)
AB480245.1(Tama) *	non-LTR	34087	322(631)	329(1492)	1832(6701)	1966(4254)
Yamato	LTR/Pao	6400	207(608)	299(1628)	1705(6094)	2087(9633)
AB480243.1(Taguchi) *	LINE/R1	32792	255(738)	302(841)	1587(7621)	1570(5069)
BmpiggyBac-MER85	ClassII^§^:TIR/piggyBac	3504	305(1379)	258(938)	1684(9442)	1456(6983)
bm228	LTR/Unknown	8077	205(473)	261(786)	1423(3304)	1760(5449)
Bmori_326.1597	LINE/Unknown	576	142(760)	148(800)	1218(6510)	2022(17937)
Gypsy-20_BM-I	LTR/ Gypsy	4469	436(1125)	261(3602)	1974(7179)	807(4194)
AB480240.1(Bm1) *	SINE/SINE	14359	337(1093)	257(1114)	1366(3521)	1466(5126)
Moriya-I	LTR/ Bel	6871	196(412)	220 (947)	1180(3113)	1350(5170)
AB480234.1(Bm1) *	SINE/SINE	18645	169(386)	183(659)	1095(5477)	1246(4397)
Kabuki	LTR/Unknown	5342	221(668)	240(2406)	1134(2787)	1002(2610)
SART1	LINE/R1	6702	77(197)	123(260)	807(1468)	1575(4629)
AB480242.1(BMC1) *	LINE/Jockey	20035	223(1024)	176(450)	1125(4006)	856(3017)
bm1222	Unknown/Unknown	1468	126(220)	160 (495)	1221(3003)	814(2988)
bm154	LINE/R1	9083	104(304)	73(171)	1224(4407)	884(1988)
AB480235.1(Kendo) *	LINE/CR1	32287	196(473)	143(368)	858(3345)	622(1317)
bm447	LTR/Pao	7110	104(140)	152 (384)	333(724)	1197(5351)
BMC1	LINE/Jockey	5091	85(152)	120(344)	765(1623)	815(2924)
bm1770	LTR/Pao	6669	96(159)	162(756)	301(646)	1089(5433)

*These contigs from the W chromosome contained TEs and generated a large amount of small RNAs around the TEs, which might be related with the transcription of the TEs.

**Total reads of TE-derived small RNAs are shown in parenthesis.

^§^TEs have been divided into two classes based on their transposition intermediate. Class I TEs use an RNA-mediated mode of transposition and are further divided into two subclasses, including LTR and non-LTR retrotransposons. Non-LTR retrotransposons are also called LINEs (long interspersed nuclear elements) and SINEs (short interspersed nuclear elements). Class II TEs use a DNA-mediated mode of "cut and paste" transposition. In *Bombyx mori*, many TEs have been identified, such as bm447, bm1770, bm1645 and SART1 [69].

The piRNAs are generated mainly from germline cells and are specifically loaded onto germline-specific Argonaute proteins-PIWI proteins [70]. Strangely, using piRNApredictor [19] and an in-house developed Perl script, a total of 54,033 reads were identified as piRNA candidates among the TE-derived small RNA reads (Table 1), which accounted for 6.64% of the total unique reads. Ping-pong pairs were defined as precise 10-nt overlaps between the sense and antisense TE-derived piRNAs and were a canonical feature of the piRNAs. We also identified 11,337 Ping-pong pairs from the 54,033 reads, with 5′-U and 10A pairing formed by 35,630 sense and antisense piRNAs. These results indicate that a single 10A-piRNA might correspond to more than one 5′U-piRNA and *vice versa* (Additional file 2: Table S5). The above results suggest that BmAgo2 may function to maintain genome stability by suppressing the activities of transposons.

### Known and novel miRNAs associated with the BmAgo2 protein

To function, miRNA must be assembled into RISC [1,71]. The Ago protein is the core component of RISC. In recent years, the isolation of miRNAs and miRNA targets through the immunoprecipitation of Ago protein complexes has become a well-characterized method to experimentally identify miRNA targets [72,73]. Therefore, large numbers of miRNAs should be contained in the BmAgo2-associated small RNAs. Of the 567 *Bombyx mori* miRNAs recorded in the miRBase (Release 18, http://www.mirbase.org/), 270 were identified in the BmAgo2-associated small RNA library, including 202 miRNAs, 66 miRNA*s, 1 miRNA-5p and 1 miRNA-3p species (Additional file 2: Table S6). Some of these miRNAs, such as *bmo-bantam*, *bmo-miR-184*, *bmo-miR-2766*, *bmo-miR-11* and *bmo-miR-100*, exhibited a clear preference for binding to BmAgo2 with a high read copy of greater than 5000.

By mapping all reads to the precursors of known miRNAs, we observed that for most known miRNA precursors, the matched reads centered around mature miRNA sequences and some miRNA* sequences (Additional file 1: Figure S4A). However, some reads showed a smear distribution for certain miRNA precursors (Additional file 1: Figure S4B), which was also observed in *Arabidopsis* Ago1 and Ago4 associated miRNA precursors. These miRNAs were not regarded as *bona fide* miRNAs, but rather as siRNAs whose "precursors" also exhibited hairpin structures [19].

Sequence variants could be involved in the editing events, SNPs and their variations. Mature miRNA variants are also known as isomiRs [74] and are usually found by small RNA deep sequencing [19,75]. A total of 44 different isomiRs, each with an abundance of at least 0.02% and representing 28 unique miRNA and 1 unique miRNA* species, were identified. The sequence length variations were not considered in this analysis, and all 44 identified isomiR species were represented by different RNA editing sites or unannotated SNPs (Additional file 2: Table S7). Interestingly, for 8 of these miRNA species, the identified responding isomiRs exhibited an abundance of 100%, suggesting that the BmAgo2 protein had a clear preference for associating with the isomiRs of these miRNAs rather than with themselves.

To identify novel miRNAs, Mireap software was used to query the unannotated and other small RNA reads. A total of 301 novel miRNA species with a read copy number of no less than 5 were identified (Additional file 2: Table S6). The precursors of these predicted novel miRNAs exhibited the typical stem-loop structure. To validate the candidate novel miRNAs, stem-loop qPCR assays were developed. The results showed that 7 of the 8 novel candidate miRNAs selected were detectable in the BmN cells, and their expression differed between the normal and the BmNPV-infected BmN cells (Additional file 1: Figure S5). A total of 301 novel miRNAs were identified from among the 892 unique reads. Of the 892 reads, 356 were derived from unannotated reads; however, the remaining 536 were derived from the annotated TE-derived small RNAs (469), piRNAs(60) and snoRNA-derived small RNAs(7) described above. The TEs and snoRNAs could generate functional miRNAs, and this phenomenon is regarded as one of the evolutionary mechanisms for miRNA formation [76-82]. Previously, 182 TE-derived miRNAs were also identified [18]. Here, our results indicate that *Bombyx* TEs could generate miRNAs associated with the BmAgo2 protein. In addition, the association between the RISC effector complex and these 301 novel candidate miRNAs also showed the biological feature of miRNAs and further validated the prediction.

Viral miRNAs can inhibit both viral and host transcripts, and usually help in maintaining the persistent infection of a virus or modulating the host antiviral response [83]. BmNPV-encoded miRNAs were first identified by J. Singh *et al.*[84], and one of these, *bmnpv-miR-1*, was shown to suppress host miRNA biogenesis by regulating the exportin-5 cofactor Ran [85]. The 4 miRNAs that were first identified did not exist among the 218 BmNPV-encoded small RNAs. However, nine novel potential viral miRNAs were identified from among the 218 BmNPV-encoded small RNAs (data not shown) using mirExplorer software [86], and these should be further validated.

### Other novel small RNAs derived from rRNAs, snoRNAs and snRNAs

Many groups have reported finding novel small RNAs derived from housekeeping non-coding RNAs, such as rRNAs, snoRNAs and snRNAs [16,87,88]. In addition to the tRFs and miRNAs, we found a large number of small RNA reads derived from rRNAs, snoRNAs and snRNAs, implying that various types of small ncRNAs associate with BmAgo2 (see GEO accession numbers GSE41841).

**Figure 4:**
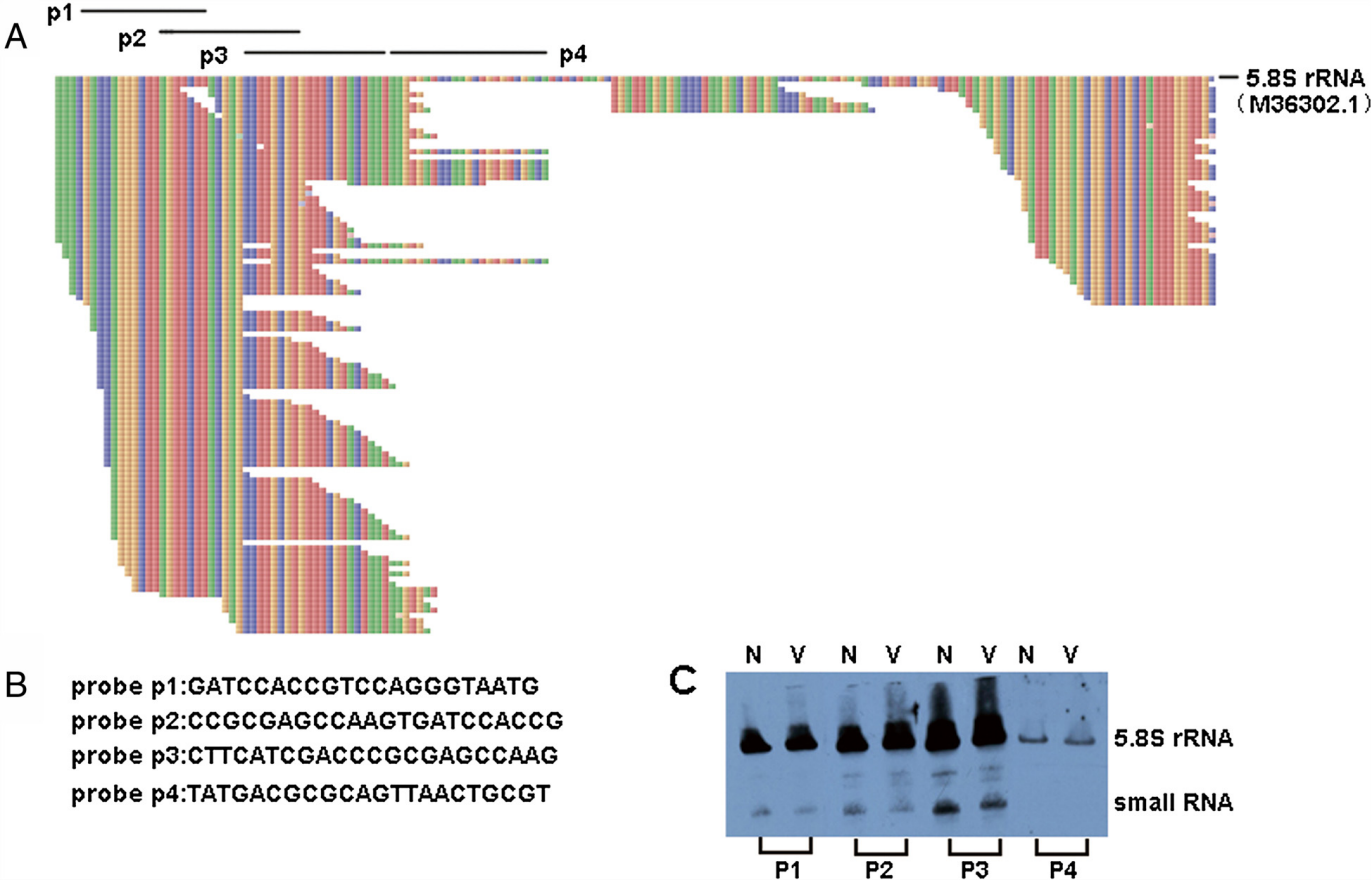
**Identification of small RNAs derived from the 5.8S rRNA of *Bombyx mori*. (A)** Mapping of reads to the 5.8S rRNA. A total of 746 reads with read copy numbers of no less than 50 were mapped mainly to the 5′ and 3′ termini of the 5.8S rRNA. The designed probes also appeared on the corresponding locations. **(B)** The sequences of the probes used to identify the small RNAs. The p1, p2 and p3 were overlapped on the 5.8S rRNA sequence. **(C)** The small RNAs derived from 5.8S rRNA were identified by Northern blotting. Their size was approximately 50nt. The small RNAs originating from the other locations on the 5.8S rRNA were not identified. N: normal BmN cells; V: BmNPV-infected BmN cells.

A total of 746 reads with copy numbers less than 50 mapped to the 5′ and 3′ termini of 5.8S rRNA. Using overlapping probes, the small RNA species derived from the 5′ termini of 5.8S rRNA were further identified by Northern blotting, and their size was found to be approximately 50nt. These small RNAs exhibited a slightly lower expression level in the virus-infected BmN cells than in the normal BmN cells (Figure 4). However, we failed to detect small RNAs originating from other locations of 5.8S rRNA by Northern blotting.

Li *et al.* previously identified 141 snoRNAs from the 50-500nt portion of the total RNA of *Bombyx mori* using RNomics and comparative genomics methods [41]. Of the 141 snoRNAs, 133 were found to generate small RNAs, mainly from 5′ and 3′ termini. A total of 4205 unique reads showed a 5′ and 3′ terminal distribution for the 133 snoRNA sequences (Additional file 2: Table S8). SnoRNAs usually function as guide RNAs to direct sequence-specific modifications of the targeted RNA. Recent work has shown that some miRNA precursors and snoRNAs share the same features, and snoRNAs can also give rise to small RNAs with miRNA-like functions, suggesting that these classes of RNAs may have an evolutionary relationship [80-82]. Like miRNAs, snoRNA-derived small RNAs are also functionally associated with the Argonaute protein [88,89]. Using mirExplorer software [86], we found that 24 of the 133 snoRNAs exhibited perfect hairpin structures similar to those of pre-miRNAs (Additional file 2: Table S9); these consisted of 607 unique reads with a total read copy number of 107,950 (Additional file 2: Table S8). The numerous snoRNA-derived molecules associated with BmAgo2 will require further study.

## Discussion

Ago proteins are the key component of the siRNA and miRNA pathway and are indispensable binding proteins for the function of many other small RNAs. For example, tRF must bind Ago2 to direct the cleavage of target RNAs, which contain a perfect complementary sequence to the tRF [16]. Using BmAgo2 polyclonal antibody, endogenous BmAgo2 was identified in BmN cells (Additional file 1: Figure S1). The Ago2 protein usually displays a Mg^2+^-dependent endonuclease activity, and Mg^2+^ provides a favorable contribution to the binding of the DNA-RNA to the Ago2 protein [90]. Our work demonstrates that recombinant BmAgo2 could exhibit an endonuclease activity and cleave the IP RNA products when EDTA is removed from the lysis buffer (Additional file 1: Figure S6), suggesting recombinant HIS-tagged BmAgo2 might exhibit the same physiological activity as endogenous BmAgo2.

Using a HIS monoclonal antibody, we successfully isolated BmAgo2-associated small RNAs by immunoprecipitation from the BmN cells. We used an RIP-seq method to obtain a total of 817,302 BmAgo2-associated unique small RNAs from the ovary-derived BmN cells, 91,092 of which we could not match to the reference genome of the male silkworm. The unmatched reads might be derived from the W chromosome or might map to potential gaps in the sequenced female genome. To date, many species of small RNAs have been identified in *Bombyx mori*, including miRNAs [24-27,29,30,91], piRNAs [35,37,38], TE-derived small RNAs [18,34] and snoRNA/snRNAs of between 50nt and 500nt [41]. In the present work, we identified BmAgo2-associated small RNAs between 18nt and 50nt in length, including the first identified tRFs, rRNA-, snoRNA- and snRNA-derived small RNAs in this species, as well as previously identified miRNAs, piRNAs and TE-derived small RNAs. Importantly, the BmAgo2 protein was found to recruit tRFs at an extremely high abundance (Figure 2B), suggesting that tRFs could function by binding the BmAgo2 protein.

We further employed Northern blotting to identify tRFs, and the results showed two obvious bands responding to the tRNAs and tRFs, respectively, suggesting that tRFs are a stable and novel class of small RNAs (Figure 3). Previous studies have shown that the ANG nuclease can generate 30-50nt long sitRNAs under conditions of stress from the anti-codon loops, and it has been reported to be a tRNA-specific enzyme [92-94]. The Bombyx 5′ and 3′tRFs identified here by Northern blotting were approximately ~33nt and ~40nt long, respectively. Northern blotting with overlapping probes further confirmed that the ~40nt 3′tRFs were generated by cleaving the anti-codon loops of the 72nt tRNAs (Additional file 1: Figure S7). These 5′ and 3′tRFs might be sitRNAs that are produced by the ANG nuclease. Our deep sequencing results further suggested that the ~33nt 5′tRFs and the ~40nt 3′tRFs could be further cleaved on their D and TψC loops by a certain nuclease, thereby generating the ~18nt 5′ truncated tRFs and ~21nt 3′trailer tRFs (Additional file 1: Figure S3), respectively. However, we failed to detect these BmAgo2-associated tRFs by Northern blotting from among the total RNAs of the normal or BmNPV-infected BmN cells, possible due to the low abundance of these tRF species. ANG and other RNases may be involved in generating these small tRF species, which can cleave tRNAs to produce ~20nt terminal tRFs [16]. Furthermore, a previous study also showed that the major determinant in the processing of terminal small tRFs by ANG and possibly by related RNases is the TψC loop within the tRNA. The ANG tRF cleavage site is known to be located between the first and second base after the GTTC motif on the TψC loop [16]. Our deep sequencing data show that a conserved GTTC domain exists at the termini of most 21nt 3′trailer tRFs, suggesting there is a potential tRF cleavage site between the GTTC cytosine and the first base after the GTTC motif on TψC loop (Additional file 1: Figure S3). The 3′trailer tRFs could not be detected by Northern blotting as they had a low total RNA abundance; however, the 3′ trailer tRFs were significantly more abundant than the 3′tRFs in the BmAgo2-associated total RNA. In the sequenced BmAgo2-associated small RNAs, the read copies of the 3′trailer tRFs were far greater than those of the 3′tRFs (Additional file 2: Table S2), suggesting that the 3′trailer tRFs preferentially associated with BmAgo2 for their functions, which might be similar to those of the siRNA pathway. For the tRNAs with a variable arm, the D loop could be cleaved at the GGCCGAGCGG cleavage site, generating the ~18nt 5′truncated tRFs (Additional file 1: Figure S3D). This result implies a possible relationship between the D loop cleavage and the variable arm during the biogenesis of 5′truncated tRFs.

The tRF biogenesis that occurs under conditions of viral stress provides an important piece of evidence about the functional role of tRFs. The present work shows that when the BmN cells were infected with BmNPV, many tRFs were generated and recruited onto the BmAgo2 protein. Therefore, these tRFs were enriched among the BmAgo2-associated small RNAs. The majority of these 5′tRFs identified by Northern blotting were expressed only in BmNPV-infected cells (Figure 3A), whereas the other tRF species identified by Northern blotting were usually expressed in both the normal and the virus-infected cells (Figure 3B), suggesting the main tRF species generated by viral stress may be the 5′tRFs. The asymmetric expression of 5′tRFs implies a relationship between the 5′tRFs and BmNPV infection in the BmN cells. Furthermore, 5′tRFs accounted for an overwhelming proportion of the BmAgo2-associated small RNAs. This was especially true for Bm-AspGTC-5′a, which accounted for 59.58% of the total BmAgo2-associated small RNAs. These observations suggest that the 5′tRFs are likely to bind the BmAgo2 protein and thus influence the BmNPV infection.

The piRNA system is thought to provide a germline defense against TE activity. A similar function was also recently noted in the siRNA pathway [51,68,95-97]. Drosophila utilizes two small-RNA systems to restrict transposon activity in its germline (mostly via piRNAs) and soma (mostly via siRNAs); in this system, Ago2 deficiency results in increased levels of transposon transcripts [95]. In mouse oocytes, the loss of Ago2 can result in decreased levels of siRNAs and increased levels of retrotransposons [97]. In *S. japonicum*, sjAgo2 strongly associated with TE-derived siRNAs and is believed to regulate transposons [18]. These observations indicate that the Ago2 protein might be involved in regulating transposons at the transcriptional level in germline cells. The BmN cell line was obtained from the *Bombyx* ovary. TE-derived small RNAs accounted for almost half of the total unique BmAgo2-associated small RNAs. The peak of their length distribution was the typical siRNA length. Therefore, we suggested that BmAgo2 could execute important functions in the maintenance of genome stability in germline cells through an RNAi-like pathway.

piRNApredictor is a software program for piRNA prediction that uses a novel 'dynamic’ algorithm with a precision of over 90% and a sensitivity of over 60% [19]. Using piRNApredictor supplemented with a perl script for screening Ping-pong pairs, 54,033 >25nt piRNA candidates and 11,337 typical Ping-pong pairs were identified from among the TE-derived small RNAs associated with BmAgo2. In addition, we further identified 56,131 >25nt piRNA candidates and 11,898 Ping-pong pairs with 17,533 sense piRNAs and 18,239 antisense piRNAs from the remaining unannotated reads (Additional file 2: Table S10). These piRNA candidates could not be mapped to the identified TEs and could be derived from some heterochromatic regions, other repeat sequences and unknown transposons in the *Bombyx* genome. piRNA is thought to defend the host genome against transposons in *Bombyx mori*[34]. Recently, an increasing number of piRNAs have been identified in *Bombyx mori*[18,35,37], and their biogenesis has been further studied, providing evidence for their "Ping-pong Circle" and biosynthesis mechanisms [37-40]. We further compared our data with the previously identified piRNAs [18,35,37]. The results showed that our data has 0.83% (909), 14.95% (16,474), 30.62% (33,733), 36.54% (40,254), 17.55% (19,335) and 17.72% (19,517) overlap (mismatch not allowed) with the TE-, BmN4-, Siwi-, BmAgo3-, ovary- and testis-derived piRNAs, respectively. This overlap serves as an indicator of the high reliability of our dataset. We first identified piRNAs associated with the BmAgo2 protein, which also contains PAZ and PIWI domains. BmAgo2 might bind to piRNA-like RNA molecules through the PAZ domain, the biogenesis of which could be similar with that of piRNAs. The observations above suggest that BmAgo2 can associate with the 20 ~ 22nt TE-derived siRNAs and the >25nt piRNA-like small RNAs during the regulation of transposon expression.

We must emphasize that many more reads did not align to previously known ncRNAs, such as miRNAs in miRBase, tRNAs, rRNAs, piRNAs, snoRNAs, snRNAs and other reported *Bombyx mori* small RNAs [41], indicating that many small RNAs remain to be discovered in *Bombyx mori*. The unannotated small RNAs associated with BmAgo2 usually exhibit a higher abundance than the miRNAs. Only 49 miRNAs had a read copy of at least 100 (Additional file 2: Table S6), but 848 unannotated small RNAs had a read copy of at least 100.

## Conclusions

We successfully overexpressed BmAgo2 under the control of the ie1 promoter in BmN cells using a recombinant BmNPV virus and used an RIP-seq approach to identify the associated small non-coding RNAs (18-50nt). Various types of small ncRNAs were found to be associated with BmAgo2, including tRNA-, TE-, rRNA-, snoRNA- and snRNA-derived small RNAs, as well as miRNAs and piRNAs. The tRFs were the most abundant class of BmAgo2-associated small RNAs. However, Northern blotting analysis showed that many of the tRFs were only expressed or significantly up-regulated in the BmNPV infected cells, implying that they act by binding BmAgo2 during the response of *Bombyx mori* to BmNPV. We also demonstrated that a significant fraction of the small RNAs associated with BmAgo2 are derived from TEs, many of which were identified as piRNA candidates. This finding suggests that they may function to maintain genome stability through the suppression of transposon activity. Finally, we also identified a large number of miRNAs associated with BmAgo2, including 301 novel miRNAs. Taken together, the above findings provide new clues for the future functional study of small RNAs during insect development and evolution.

## Methods

### Cell line

The BmN cell line, maintained in our laboratory and derived from the silkworm ovary, was cultured in Sf-900 II SFM medium (Gibco BRL) supplemented with 10% (v/v) fetal bovine serum (Gibco BRL) at 27°C.

### Expression of BmAgo2 using the Bacmid expression system and Western blotting analysis

BmAgo2 was expressed using the Bacmid system under the control of the ie1 promoter enhanced with hr5 of *Autographa Californica* nucleopolyhedrovirus (AcNPV). The recombinant Bacmid vector harboring the *BmAgo2* gene was previously constructed by us and was preserved in our laboratory [61]. In this Bacmid, the immediate early ie1 promoter and the hr5 enhancer were introduced and used to drive the expression of BmAgo2. BmN cells were infected with the constructed recombinant virus at an MOI of 10 and cultured at 27°C. After 17 h, the cells were harvested and washed twice with PBS (pH 7.4). The cells were then lysed in cold lysis buffer (50 mM Tris, pH 8.0, 150 mM NaCl, 5 mM EDTA, 0.5% Nonidet P-40) with freshly added 1 mM DTT for 30 min. The expressed HIS-BmAgo2 protein was analyzed using the previously described method of Western blotting with the mouse Anti-His_6_ monoclonal antibody (Roche) (1:2000) [98]. Briefly, cell extracts with over-expressed BmAgo2 were separated on an SDS-PAGE gel and transferred to a PVDF membrane (Millipore). The membrane was blocked with 5% BSA for 90 min at room temperature. Anti-His_6_ antibody was used for the detection of HIS-BmAgo2. The HRP-conjugated goat mouse IgG (1:10,000) was used as the secondary antibody, and the signal was detected using the DAB kit (Gersion).

### Co-immunoprecipitation

Cells infected with recombinant virus were lysed in an ice-cold lysis buffer (50 mM Tris, pH 8.0, 150 mM NaCl, 5 mM EDTA, 0.5% Nonidet P-40) with freshly added 1 mM DTT, 200 U/ml RNase inhibitor (Promega) and a protease inhibitor cocktail (Roche) for 30 min at -80°C. After dissolving at room temperature, centrifugation was performed at 12000 rpm at 4°C for 10 min. The supernatant was first mixed with 40 μl of protein G magnetic beads (Millipore) and incubated at 4°C for 3 h with gentle agitation (Mock). After immobilizing the magnetic beads with a magnet, the supernatant was removed and subsequently mixed with 5 μl of Anti-His_6_ antibody or normal mouse IgG for 3 h at 4°C with gentle agitation. Then, 40 μl of the protein G magnetic beads were added and continually incubated for 3 h at 4°C. The immunoprecipitate was washed five times in lysis buffer and separated into two portions, one for the RNA isolation as a sample for the following experiment and another for the Western blotting to identify the immunoprecipitation of BmAgo2. Normal Mouse IgG was used as a negative control in the immunoprecipitation procedure. The eluted proteins were separated on SDS-PAGE gel and identified by Western blotting with an Anti-His_6_ antibody (1:2000) and the ECL Plus Western Blotting Detection System (GE Healthcare).

### RNA extraction

The total RNAs were extracted from the immunoprecipitate of BmAgo2 using TRIzol reagent (Invitrogen) according to the manufacturer’s protocol, the concentration and absorbance of which were determined using a Nanodrop ND-1000 spectrophotometer. The total RNAs were subsequently loaded onto a 15% denaturing polyacrylamide gel (PAGE) containing 8 M urea. They were electrophoresed and then stained with 1 μg/μl ethidium bromide.

### RNA-seq

A small RNA library was constructed using the total RNAs extracted above. Briefly, the RNAs were size-fractionated on a 15% polyacrylamide gel, and the 18-50nt fraction was collected. The collected small RNAs were ligated with 5′ and 3′ Illumina adaptors and subsequently used as a template to synthesize first-strand cDNA. The cDNA was amplified by PCR with the Illumina small RNA primer set and sequenced on the Illumina HiSeq 2000 at BGI (Beijing Genomics Institute, Shenzhen, China). We failed to construct a small RNA library for the negative control due to the low concentration of total RNAs extracted from the negative control of the immunoprecipitate.

### Small RNA sequence analysis

Adaptors, low-quality tags and contaminants were removed from raw reads produced by Solexa sequencing to produce the clean total reads. All identical reads were counted and merged as unique reads. The length distribution of the clean total reads and unique reads were then summarized. These unique reads with read counts were mapped to the *Bombyx mori*[62,63] and BmNPV genome [99] using Bowtie or SOAP2 software with 0 or 2nt mismatch [100,101]. Afterwards, the clean reads that perfectly mapped the genome were annotated into different categories according to the standard bioinformatics pipeline designed by GBI. Briefly, by comparing the clean reads with those in existing databases and picking out the overlap on genome location between our data and the databases using BLAST or other programs developed by GBI, the small RNA reads were annotated into different categories. These categories included miRNAs as well as TE-, tRNA-, rRNA-, snoRNA- and snRNA-derived small RNAs. The remaining reads were then used to predict the novel miRNAs and to base edit the potential known miRNAs. The >25nt TE-derived small RNAs and remaining unannotated reads were selected for probing piRNAs using the piRNApredictor program [19] and Perl script developed by us. The reads that perfectly mapped to the BmNPV genome were used to predict the BmNPV-encoded miRNAs using mirExplorer software [86]. Finally, the remaining and unmapped reads were labeled as unannotated small RNAs. The whole process is shown in Additional file 1: Figure S8.

Datasets downloaded for the bioinformatics analysis above are listed as follows: *Bombyx* miRNAs was derived from miRbase 18.0 (http://www.mirbase.org). Silkworm ncRNAs used for the annotations, including the tRNA, rRNA, scRNA, snoRNA and snRNA annotations, were obtained from NCBI and Rfam (http://www.ncbi.nlm.nih.gov and http://rfam.sanger.ac.uk); 1476 annotated silkworm TE sequences were retrieved from NCBI, NIAS DNA Bank (http://www.dna.affrc.go.jp) and the BmTEdb database (http://gene.cqu.edu.cn/BmTEdb) and 1668 ReAS clones were obtained from the BmTE Library (http://sgp.dna.affrc.go.jp/pubdata/genomicsequences.html). The obtained silkworm TE sequences were merged using CD-HIT software [102]. Some contigs containing TEs from the W chromosome were also included in our TE datasets to probe TE-derived small RNAs, which might be related to the transcription of the TEs. The BmN4-, Siwi-, BmAgo3-, ovary-, testis- and TE-derived piRNAs used for the comparative analysis were retrieved from DDBJ (http://www.ddbj.nig.ac.jp/) and the Supplementary materials according to the references [18,35,37].

### Northern blotting

The oligonucleotide probes were labeled with the DIG Oligonucleotide Tailing Kit (Roche) according to the manufacturer’s instructions. Illustra ProbeQuant G-25 Micro Columns (GE Healthcare) were used for the purification of probes after labeling.

Northern blotting analysis was performed on the total RNA extracted from the normal BmN cells and the BmN cells infected with the BmNPV virus. The total RNAs were mixed with Gel Loading Buffer ІІ (Ambion), heated to 95°C for 3 min and separated on 15% denaturing TBE-Urea gels. Next, the RNAs were transferred onto the charged nylon membranes (Millipore), which were then subjected to UV cross-linking, pre-hybridized at 42°C for 2 h using the PerfectHyb Plus Hybridization Buffer (Sigma) and hybridized with probes overnight at 37°C. After hybridization, the membranes were rinsed and washed four times (twice at 25°C for 15 min using 2× SSC and 0.1% SDS, twice at 37°C with 0.5× SSC and 0.1% SDS). Signals were detected with a DIG Luminescent Detection Kit (Roche) according to the manufacturer’s protocol and were finally exposed to X-OMAT BT film (Kodak).

### Stem-loop qRT-PCR

The novel miRNAs predicted were further identified by miRNA-specific stem-loop quantitative real-time PCR using the mirVana™ qRT-PCR miRNA Detection kit (Ambion) as described previously [103]. The top 8 novel miRNAs with the highest read copy number were selected for qRT-PCR identification.

## Abbreviations

ncRNAs: non-coding RNAs; BmAgo2: *Bombyx mori* argonaute2; tRFs: tRNA-derived fragments; miRNAs: microRNAs; siRNAs: small-interfering RNAs; piRNAs: piwi-interacting RNAs; IP: Immunoprecipitation; RIP: RNA immunoprecipitation; TE: Transposable element; RISC: RNA-induced silencing complex; AcNPV: *Autographa Californica* nucleopolyhedrovirus; BmNPV: *Bombyx mori* nucleopolyhedrovirus; PAGE: Polyacrylamide gel.

## Competing interests

The authors declare that they have no competing interests.

## Authors’ contributions

ZMN performed the experimental design and bioinformatics analysis and drafted the manuscript. FZ performed the IP and the construction of the small RNA libraries. DL, ZBL and JC performed the Northern blotting analysis. YL and JHS performed the stem-loop qRT-PCR analysis. QS performed the IP. WY was responsible for the cell culture. WPZ and CYJ performed the expression study of BmAgo2. YHY performed the bioinformatics analysis. JMY and YFJ designed the small RNA sequence analysis. YZZ was responsible for the experimental design and revised the manuscript. All authors approved the final version of the paper.

## Supplementary Material

Additional file 1 Supplementary figures contain 8 figures. Click here for file

Additional file 2 Annotation and analysis of small RNAs associated with BmAgo2 contain 10 tables. Click here for file
