# Supplementary material for: RIP-seq of BmAgo2-associated small RNAs reveal various types of small non-coding RNAs in the silkworm, Bombyx mori
Source: BMC Genomics. 2013 Sep 28;14:661. doi: 10.1186/1471-2164-14-661 (PMC3849828; doi:10.1186/1471-2164-14-661)
Supplement: Additional file 1 — Supplementary figures contain 8 figures. [file 1471-2164-14-661-S1.doc]

**Additional file1**


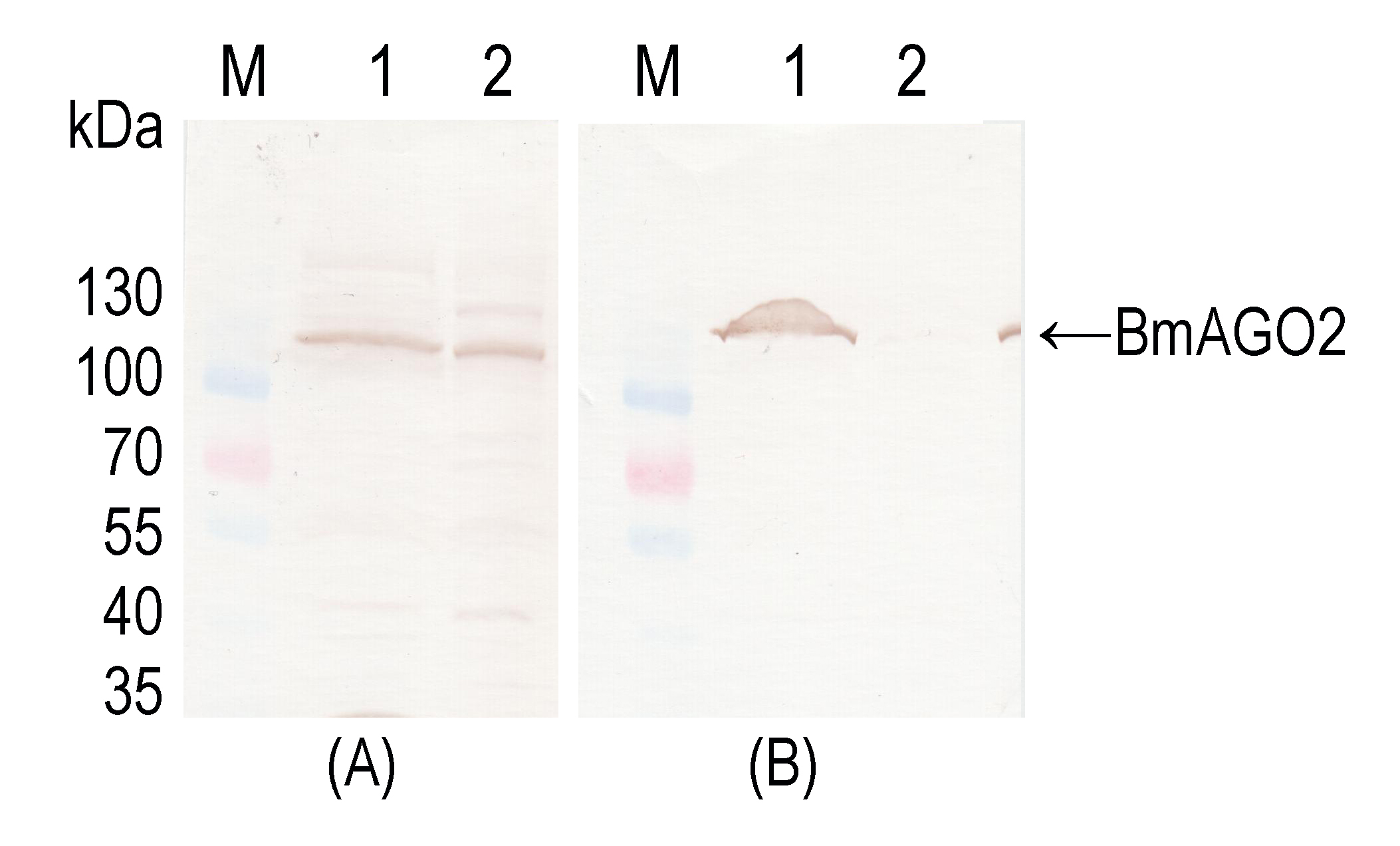


**Figure S1 Western blotting of BmAGO2 expressed using the ie1-Bacmid expression system in BmN cells.** (A) Detection using the BmAGO2 polyclonal antibody. (B) Detection using the His monoclonal antibody. M: Pre-stained Marker; 1: BmN cells infected with the ie1-vBacmid-*BmAGO2* recombinant virus; 2: normal BmN cells. Samples of 30 µg total protein each were size-fractionated by SDS-PAGE and transferred to a PVDF membrane.


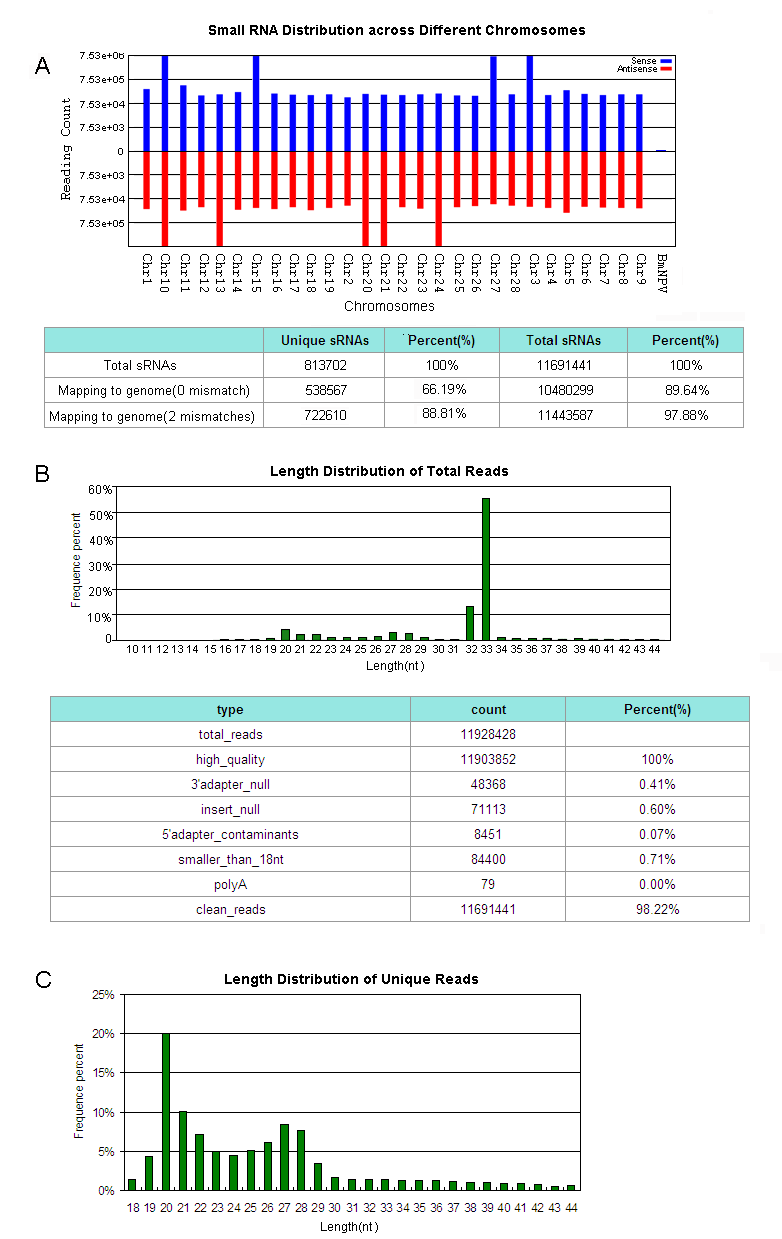


**Figure S2 Overview of BmAGO2-associated small RNAs library.** (A) Genomic distribution of unique small RNAs. There was no chromosome distribution preference. (B) Length distribution of total small RNAs. The total BmAGO2-associated small RNAs were dominated by ~20nt, ~27nt and ~33nt long species, with the population of ~33nt small RNAs being much larger than that of the ~20nt and ~27nt ones. (C) Length distribution of unique small RNAs. Interestingly, the unique BmAGO2-associated small RNAs were dominated by the ~20nt and ~27nt long species.


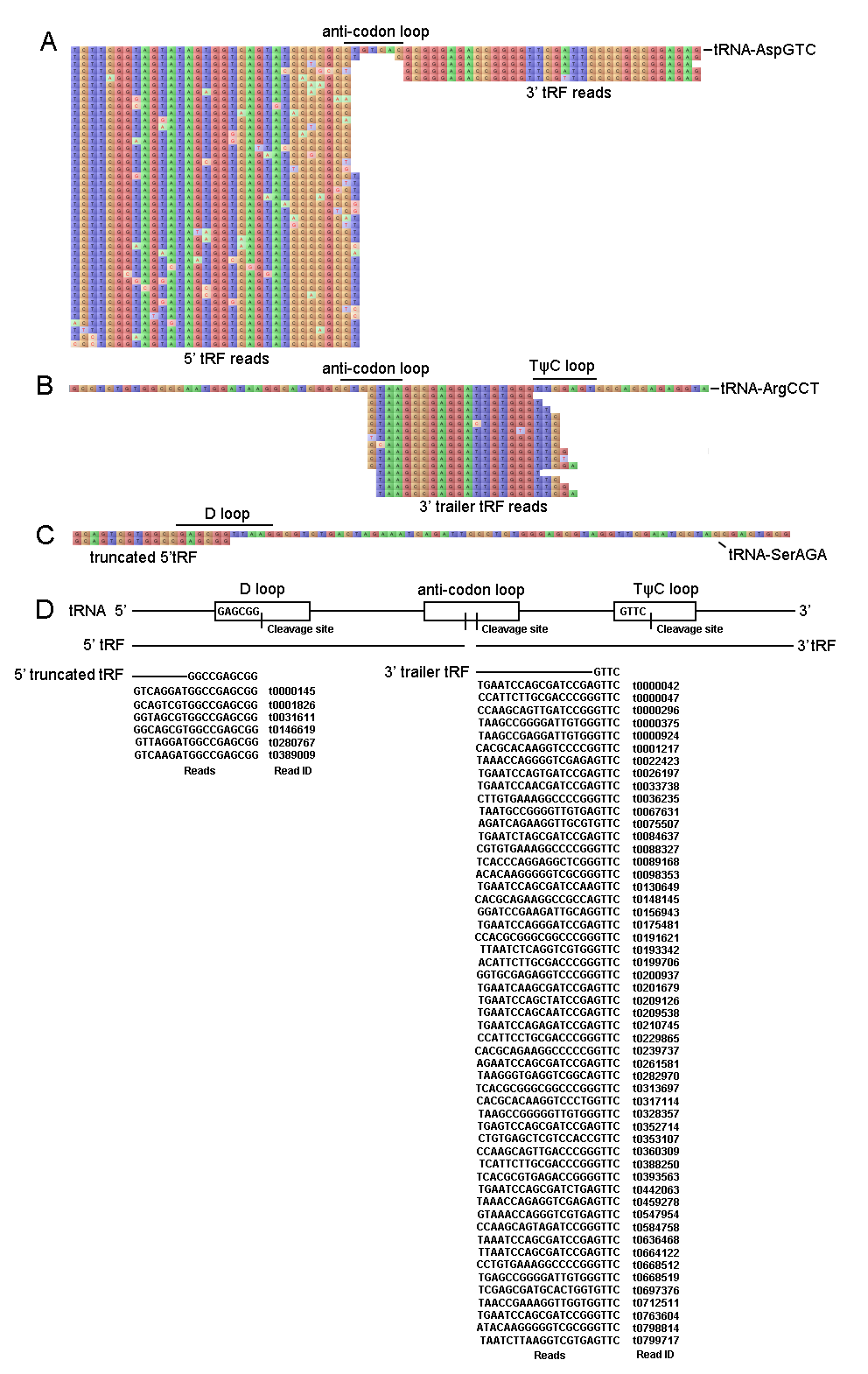


**Figure S3 tRFs might be derived from tRNAs though the cleavage of the D loop, anti-codon loop and TψC loop.** (A) tRNA-AspGTC was cleaved on the anti-codon loop, generating 5’tRFs and 3’tRFs. (B) tRNA-ArgCCT was cleaved on the anti-codon and TψC loop, generating 3’ trailer tRFs. (C) tRNA-SeAGA was cleaved on the D loop, generating 5’ truncated tRFs. (D) The cleavage sites on the D loop and TψC loop were located at the end of conserved GGCCGAGCGG and GTTC motifs, respectively. Therefore, the 5’ truncated tRFs and 3’ trailer tRFs usually contained the conserved GGCCGAGCGG and GTTC motifs, respectively. The D loop cleavage only occurred in tRNAs containing the variable arm, suggesting a possible relationship between the D loop cleavage and the variable arm.


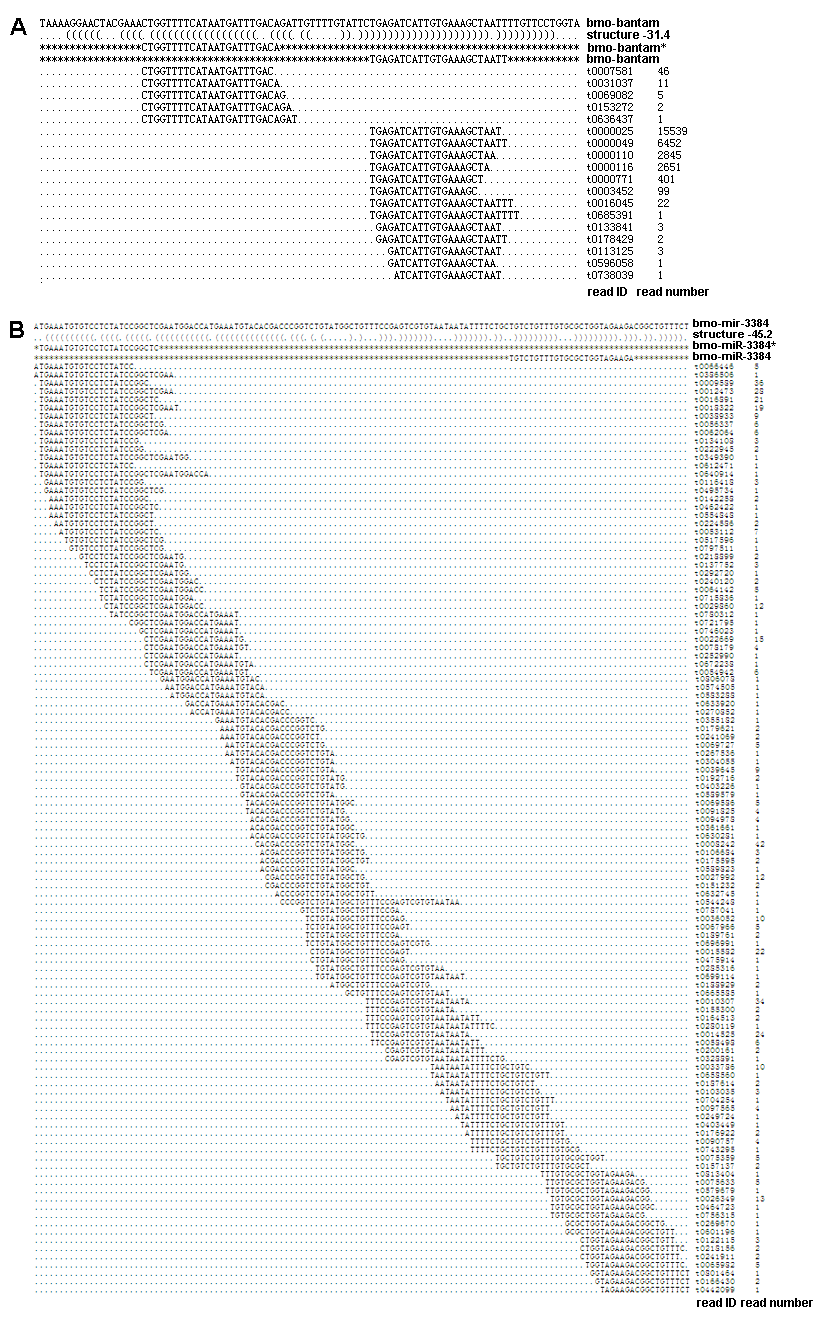


**Figure S4 Mapping of reads on the miRNA precursors.** (A) Centered distribution of reads around *bmo-bantam* and *bmo-bantam**. (B) Smeared distribution of reads on *bmo-mir-3384*. The smeared distribution was different than that of *bmo-mir-3384* that was previously recorded in miRBase (MI0014445). The bases outside of the reads on miRNA precursors are presented by dots.

**Figure S5 Stem-loop qRT-PCR identification of novel miRNAs predicted by Mireap software from the BmAgo2-associated small RNAs**. The known miRNAs *bmo-miR-133* and *bmo-miR-184* showed a very low and high expression level in BmN cells, respectively, which was shown via a miRNA microarray assay (77) and deep sequencing. Therefore, these two miRNAs were used as the negative and positive controls, respectively. Compared with the control miRNAs, 7 of the 8 selected novel miRNAs were identified, and their expression differed between the normal and the BmNPV-infected BmN cells.


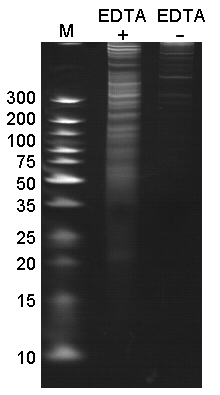


**Figure S6 The endonuclease activity of the recombinant BmAgo2.** The recombinant HIS-BmAgo2 could exhibit an endonuclease activity and cleave the IP RNA products when the EDTA was removed from the lysis buffer (EDTA-). The EDTA could inhibit the endonuclease activity as an enzyme inhibitor (EDTA+). M: DNA marker.


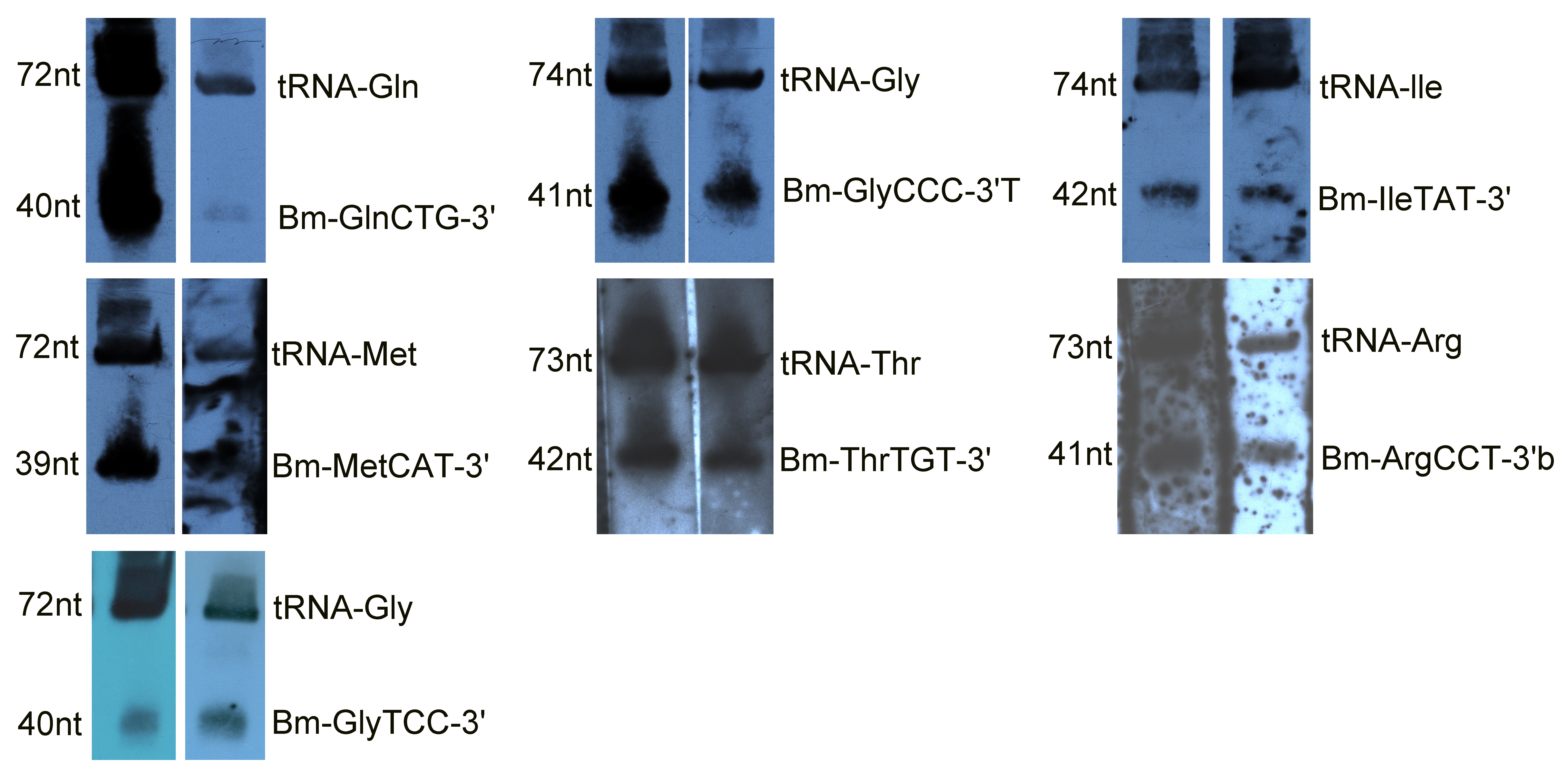


**Figure S7 Identification of 3’tRFs by Northern blotting using phased probes in the BmNPV-infected BmN cells.** Each of the selected 3’tRFs was hybridized with two overlapping probes, which covered the complete 3’tRF sequence. Two bands of the same size were observed using the two overlapping probes, one of which was located on the anti-codon loop. This suggests that the ~40nt 3’tRF might be generated by cleaving the anti-codon loop of the ~72nt tRNA. Another ~32nt 5’tRF was yielded at the same time.

**Solexa reads**

**Mapped reads**

**Unmapped reads**

Standard analysis

**High quality reads**

**Mapping reads to genome**

Clean up too small reads and contaminations formed by adaptor-adaptor ligation

Trim 3’ adaptor

Filter low quality reads

**Full-length**

**small RNA reads**

**Common/Specific**

**sequences**

**Length**

**distribution**

**tRNA-, rRNA-, snoRNA-, snRNA-derived small RNAs**

**Known miRNAs**

**BmNPV-encoded small RNAs**

**Unannotated small RNAs**

**TE-derived small RNAs, piRNAs**

Annotation

**Differential expression**

**Target prediction**

**Cluster analysis**

**Novel miRNAs**

**Potential miRNA with seed edit**

**GO enrichment and KEGG pathway analysis**

**Figure S8 Flow Chart of the small RNA sequences analysis**
